# Unpalatable Truths: Food and Drink as Medicine in Colonial British India

**DOI:** 10.1093/jhmas/jry011

**Published:** 2018-03-13

**Authors:** Sam Goodman

**Affiliations:** Weymouth House, Bournemouth University, Fern Barrow, Poole, Dorset, United Kingdom of Great Britain and Northern Ireland, BH12 5BB

**Keywords:** Medicine, health, India, Victorian, Empire, Alcohol

## Abstract

This article considers the significance of eating and drinking within a series of diaries and journals produced in British colonial India during the Indian Rebellion of 1857. The discussion of food and drink in this context was not simply a means to add color or compelling detail to these accounts, but was instead a vital ingredient of the authors’ understanding of health and medical treatment. These texts suggest a broader colonial medical understanding of the importance of regulating diet to maintain physical health. Concern with food, and the lack thereof, was understandably a key element in diaries, and in the eyewitness accounts kept by British soldiers, doctors, and civilians during the rebellion. At a narrative level, mention of food also functioned as a trope serving to increase dramatic tension and to capture an imagery of fortitude. In references to drink, by contrast, these sources reveal a conflict between professional and lay opinions regarding the use of alcohol as part of medical treatment. The accounts show the persistent use of alcohol both for medicinal and restorative purposes, despite growing social and medical anxieties over its ill-effects on the body. Close examination of these references to food and drink reflect the quotidian habits, social composition, and the extent of professional and lay knowledge of health and medicine in colonial British India.

Colonial India has always occupied a place at the extremes of the British imagination. As an array of memoirs, journals, travelogues, and diaries record, the Raj was the site of privileged splendor and grand dinners attended to by dutiful wallahs.^1^ Yet, India was equally a place of constant hardship and deadly risk, arising from illnesses such as cholera, typhoid and smallpox, as well the natural hazards of heat, snakebites, and the constant plague of flies and mosquitos. These extremes in experience were not, however, unconnected, and many Anglo-Indians saw the risks as a price worth paying. Lieutenant Walter Campbell wrote of how many of his men viewed India as “hopeless banishment…from whence, if they escaped an early death, they would return with sallow cheeks, peevish tempers, and ruined constitutions.”^2^ In Campbell’s “romantic imagination” though, India was also “a land of sunshine and perfume” full of spectacle and other delights to the senses.

One link between these competing visions of India is their corporeality. Such perceptions of colonial life support E. M. Collingham’s oft-quoted statement that the British experience of India was “intensely physical,” because of the environmental contrast between Britain and India, but also because of the ever-present specter of illness and death.^3^ The British experience of India, and its representation in print cultures, has always involved medicine and health. Ill health while in India was a recurring theme in the autobiographical writings of the period, while a parallel library of medical pamphlets and guides provided advice on how to avoid or remedy sickness. Much of the history of medicine in British India has drawn its focus on bodily health, exploring, as Mark Harrison notes, the linked concepts of “normative ecology” and “medical topography,” with attention to how varying physiological types responded to the Indian environment, and to traditional aspects of place, such as “airs, waters, [and] customs.”^4^ Under the ever-present threat of epidemic disease, and of the physical maladies likely to befall a European body in the spaces of the subcontinent, the preservation of life in British India depended, as Collingham argues, on quotidian “bodily practices,” such as grooming, dress, washing, eating, and drinking.

Narrative accounts from the colonial period are invariably sensory documents, a testament to what their authors felt, consumed, contracted, and suffered. The regularity with which eating and drinking appear as subjects, as well as the minute detail in which such activities were typically described, suggests that they were intrinsic to the British subject’s experience of India. Luxurious life in India came relatively cheaply. Private soldiers and non-mercantile residents (such as schoolteachers, the clergy and others) were able to engage servants or pay native Indians for the regular procurement of supplies.^5^ Food and drink, were also important to narrative accounts of the crisis of the Indian Rebellion of 1857-58, when the lack of supplies posed a very tangible threat to health and to the successful treatment of illness.

In the diaries and journals of British colonial India, food and drink did not simply add color or compelling detail, but were vital to the authors’ understanding of health and medical treatment. These texts suggest a broader colonial medical understanding of the importance of regulating diet to maintain physical health.^6^ Concern with food was a key element in diaries, and in the eyewitness accounts kept by British soldiers, doctors, and civilians during the rebellion. At a narrative level, mention of food increased dramatic tension and captured an imagery of fortitude. The accounts also show the persistent use of alcohol for medicinal purposes, despite growing social and medical anxieties over its ill-effects on the body. In references to drink these sources reveal a conflict between professional and lay opinions regarding the use of alcohol as part of medical treatment. Alcohol is a constant presence in accounts of life in British India. Despite medical opinion to the contrary, alcohol is portrayed as a boost to morale or as a replacement for food. Alcohol also appears as a preventative or restorative in the treatment of illnesses, as well as the general maintenance of health, with variable results.

## Diaries and Everyday Experiences of British India

This paper examines how diarists of the siege of Lucknow recorded the use of food and drink as medicine. Before discussing the diaries, it is necessary to set their accounts within the context of the British position in mid-nineteenth-century India. As historians Bernard Porter and Andrew Thompson have illustrated, it is difficult to ascertain the extent to which the general public in Britain was aware of or actively interested in the nation’s colonial Empire in India during the nineteenth century.^7^ Although India eventually became the “jewel in the crown” of the British Empire, in the eighteenth century management was largely in the hands of the East India Company and was as much a commercial enterprise as it was part of the empire.^8^ The number of Europeans in India was low; Richard Holmes notes that British and other Europeans accounted for only around 40,000 inhabitants in a population of one million in 1837.^9^ Nevertheless, there existed enough of an interest in India to create a culture of publication in Britain in which the reminiscences and experiences of returning travelers, soldiers, or statesmen could find both publisher and audience.^10^

Public interest in Indian affairs rose significantly during and after the Indian Rebellion of 1857. The rebellion was a watershed moment in British rule in India. The causes were complex, and its roots lay in a number of factors related to native Indian anxieties over religious conversion, the disenfranchisement of Indian landed-classes, as well as continued British territorial expansion and demands on native troops to serve overseas. The flashpoint for disorder, however, began over the issue of a new rifle cartridge rumored to be greased with both pork and beef tallow, thus making it offensive to both Hindu and Muslim Sepoys.^11^ The result was a rebellion of native military forces across the central plains of India in the summer of 1857, which gathered popular support from local dignitaries such as Nana Sahib and the Rani of Jhansi.

The ferocity of the uprising, felt particularly at Dehli, Lucknow, and Cawnpore, and the widespread support that it garnered shocked the majority of British residents, who believed that their presence on the subcontinent was an effort to bring prosperity not only to Britain but to India too. British residents in India during this period believed fervently in the so-called “civilizing mission” of Empire, replacing the mercantile ideals of the East India Company (EIC) with something altogether more genteel and palatable to Victorian sensibilities.^12^

The exceptional nature of the rebellion, including the battles and sieges themselves, fueled an appetite for diaries in the rebellion’s aftermath.^13^ Unlike the accounts of previous Indian campaigns, such as the Anglo-Sikh War of 1845-6, the Indian Rebellion saw few set-piece battles. Sieges, such as at Cawnpore, Dehli, Agra and Lucknow, involved significant numbers of civilian volunteers and their families, including women and children. Claudia Klaver notes the relative novelty of this situation, and views the presence of women as directly related to the “civilizing mission.” As the British establishment in India grew to become more concerned not so much with direct rule as with the transmission of British values, greater numbers of women joined their husbands and provided visible examples of British domesticity and marital harmony. Many of these women found themselves eyewitnesses to the rebellion and their diaries provide a different perspective on events.^14^

The nature of the many sieges meant that eyewitnesses would be drawn from all ranks and branches of military and civil service, but also from the range of classes represented in the stations. As Klaver observes, the position of female diarists within the social hierarchy directly corresponded to that of their husband’s position within the military chain of command, or the nature of their employment if civilians.^15^ The diaries examined here represent this range of classes. At Lucknow, Julia Inglis, wife of Brigadier John Inglis, was the “burra mem” or “first lady” of the garrison, followed by the wives of other senior officers such as Adelaide Case; Maria Germon, whose husband was an officer in a Crown regiment, Katherine Polehampton, wife of the garrison’s Reverend Henry Polehampton, and Georgina Harris, married to Chaplain James Harris, were next; through finally to Colina Brydon, married to surgeon William Brydon, and Katherine Bartrum, married to a junior officer in a Native Infantry regiment.^16^ The range of available perspectives in turn served to enhance the sensational and vicarious nature of the diaries produced in the rebellion’s aftermath. As such, these documents embody a variety of literary and expressive modes. They are positioned as objective records of events (validating the origins of the diary form in its modern sense as a “true account”) but also as personal views that trade on the identity of the author as a means of engaging and interesting the reader and further as sensational narratives, subject to the influence of literary devices to lure and hold the attention of the reader.^17^

## Food, Health and Medicine

As well as containing the sensational details of siege warfare, the diaries produced at Lucknow and elsewhere included everyday occurrences, such as the consumption of food and drink. Almost all narratives from the Indian Rebellion, as well as Indian memoirs in general, record food and drink. Food and drink served a variety of narratological functions. First, a record of food and drink consumed (along with other, sometimes mundane details such as dining companions, prices paid, and weather) is for the author’s own reminiscences. Such records befit a daily diary format and would also have been of interest to relatives back in Britain. Second, recording the more outlandish or luxurious meals conformed to a tendency to exoticize India and its culture and call attention to its various insanitary conditions.^18^ Occasionally, a diarist included details of food and drink out of sheer boredom. On long journeys, or in tedious postings, numerous diarists noted how they came to obsess over meals. For example, in William Russell’s memoir *The Indian Mutiny: A Diary of the Sepoy Rebellion* (1860), Russell apologized for the inordinate amount of time he spent detailing his meals, but argued in his defense that given the confines of the “world” of the ship passengers must take amusement where they can.^19^

Diary records of food also show how eating and drinking were seen as key to maintaining health while overseas. The association between food and the maintenance of health was a concern of Anglo-Indian doctors, dieticians and the British authorities throughout the duration of colonial rule and a wealth of sources and information on the relationship between food and health was publicly available throughout the nineteenth century. Travel guides or medical handbooks such as Edmund C. P. Hull’s *The European in India: or Anglo-Indian’s Vade-Mecum, with Medical Guide for Anglo-Indians* (1874), recommend eating “plenty of good wholesome food” but to “be at the same time moderate” taking care to consume less “animal food” and more vegetables as a result of the climate.^20^ In cases of actual sickness though, such guides emphasized the opposite and extolled the benefits of nourishing animal foods such as stews and broth as part of treatment, a prescription that largely remained unchanged for the bulk of British rule. Writing on cases of “Pucka Fever” in 1793, John Peter Wade MD, of the Honorable EIC’s Bengal Establishment, argued for the administering of chicken broth, lime juice, kid, fowl, bread pudding and rhubarb as a means of restoring strength and inducing bowel-movement.^21^ Nearly a century later, Hull’s book (in a detailed section on health written by R. S. Mair, “Late Deputy Coroner of Madras”) reiterated near-identical advice regarding a range of afflictions from diarrhea to “diseases of the liver” with the addition of beef-tea, arrowroot, sago, and castor oil.^22^

As well as this general context of medical advice on diet, the relationship between health and the provision of good quality food was also a concern of the EIC and the British military. As the India Office archives and a range of literature within the medical history of India suggest, military authorities paid close attention to the provisioning and supply of their troops, and took various steps to ensure healthy diet across the nineteenth century so to avoid decrease in fighting strength or losses to physical wastage.^23^ Indeed, the maintenance of the good health of the military was vital to the preservation of imperial power, especially in colonies such as India where the mortality rate of European troops was likewise high and the supply of replacement men both expensive and intermittent. For example, an extensive report from 1883-5 examined the provisioning of troops in various stations and whether their commanding officers believed rations to be sufficient for the work or service in which they were engaged.^24^ From this broader picture, the Surgeon General, A. D. Home (himself a veteran of Lucknow) made various recommendations for changes in rations in order to keep men from buying food and drink in the native bazaars, long thought to expose them to sickness as a result of poor quality of food and sanitation. Another policy involved the “seasoning” of troops by first posting them to colonial territories in which they might adjust to the change in living conditions. While predominantly concerned with gradually improving their tolerance of the Indian climate, Erica Wald argues that the process of seasoning also included acclimatization to the change in diet prompted by posting to the subcontinent.^25^

Given the predominance of medical officers in situ at Lucknow and the duration of service of many of its key residents, it is evident that these diarists possessed knowledge and understanding of the relationship between health and diet.^26^ Indeed, in the course of many of the narratives from Lucknow the importance of consuming the correct kinds of food for the maintenance of good health became an oft-repeated imperative. However, at the outset of the disturbance, there was little change in routine other than some prudence and forward planning in terms of storing food in case of a protracted siege. Maria Germon wrote on June 24^th^, six weeks after the beginning of the rebellion at Meerut on May 10^th^, that she visited Mrs. Fayrer (wife of Assistant Surgeon Joseph Fayrer) “and saw all her stores in case of a siege. Rice and flour all in large earthen jars that put in mind…the forty thieves…in Ali Baba.”^27^ Even after the siege began in earnest and the majority of the native servants escaped on the 1^st^ July, Germon did not record any great hardship; she noted that her kitmatgur (a type of butler) remained, along with some other servants, who continued to cook for their employers, and she mentioned preparing her own tea to accompany a chota hazree (breakfast) of “roast mutton, chupatties, rice and jam.”^28^

As revealed by a number of accounts, breakfast and dinner were considered the two most vital meals of the day, in both a physiological and psychological sense. Germon and Inglis both emphasized the importance of breakfast, and explained how their husbands joined them to eat, discuss the progress of the siege, and to occasionally read psalms and prayers.^29^ Breakfasts were often described as large meals, corresponding to the advice of contemporary medical publications. For instance, Surgeon Major Joshua Duke’s *Banting in India with Some Remarks on Diet and things in General* (1885), based on his own service experience beginning in the late 1850s, recommended two breakfasts: a light meal before leaving quarters, and then a larger one at approximately 9 or 10 am.^30^ Duke stated that heavy physical work would likely occur before the full heat of the sun so it was vital to ensure sufficient food has been taken to support such activity. Similarly, residents made explicit effort to dine with one another throughout the duration of the siege, despite the apparent dangers of gathering together in such a fashion: Colina Brydon and Julia Inglis both describe the occasion in which Dr Brydon is shot while dining.^31^ A tendency to gather for dinner in this fashion reinforced the ritual importance of eating and drinking significant to the experience of the rebellion, but also, as Collingham argues, to the wider British experience of India.^32^ While for Collingham it represents a means of enforcing conformity, within the context of the siege meals acted as a routine that helped to provide a stable anchor in the midst of exceptional events. For military wives like Germon, Inglis and Brydon, the formal aspect of shared dining became a fleeting moment of familiar and comforting order in the midst of great physical and social upheaval. Compared to breakfast, the food served at dinner was often lighter, and in much smaller portions, again conforming to contemporary medical advice.^33^ Germon’s descriptions of her and her husband’s dinners were often comparatively slight; barring the occasional surfeit of supplies (such as a tin of salmon and a “roly pudding of suet and attah” on the twenty-first August, which they did not share with the other residents), dinner could consist of a ration biscuit and a glass of port or sherry.^34^

As time progressed, the record of what the various members of the garrison ate came to reinforce the broader rebellion narrative of British endurance and self-reliance. For example, previously well-to-do, middle-to-upper class women were forced to rely on their own resources. Many accounts suggest that the women and the garrison tended to regard these domestic duties and new hardships with good humor, even pleasure, especially while supplies were relatively plentiful and the act of cooking (usually not engaged in except by the poorest of Europeans in India given the cheapness of servants) was a novelty. For example, Emily Polehampton rather gaily mentioned the difficulty Katherine Bartrum experienced tenderizing and then cutting up a recently slaughtered gun bullock with nail scissors. The women took great care to record their meals, often with pride at having been able to produce edible food at all.^35^ Colina Brydon, when not on guard duty or tending to the sick, turned her hand to baking, noting with a sense of achievement in her diary that “some bread I made last night turned out rather well.”^36^ Cooking and the provision of food in order to maintain strength and health of their husbands and families formed part of the ways in which these women had the ideals of Victorian domesticity thrust upon them as a consequence of the siege.^37^ At the same time, however, the framing of individual narratives also exerts an influence on the presentation of such details, and can vary in tone dependent on the circumstances of its composition. For instance, Katherine Bartrum’s siege diary is entitled *A Widow's Reminiscences of the Siege of Lucknow*, and unlike Inglis or Germon (whose husbands survived the siege) she was compelled by her widowhood to publish, lending her account an altogether more desperate air.^38^ On the 1^st^ of July, a month before Polehampton described Bartrum’s skill with nail scissors, her own account recorded that: “meat, peas, atta, rice, and sea biscuits, were put together into a saucepan with some water and made into a stew; but as the saucepan was of copper and could not be relined during the siege, the food when it was turned out was often perfectly green - hunger alone could make it enjoyable.”^39^ Likewise, she made other references to the danger her child, at this point little over a year old, experienced as a consequence of being deprived of milk and other sources of nourishment. Along with this tension, Germon also acknowledged the exceptional status of the siege with regard to diet as well as a little comedy, mentioning that in spite of hunger and the positive verdict of her dining companions she “could not be induced to taste” Dr Fayrer’s sparrow curry, for which he had personally shot 150 birds.^40^

As the siege continued into its third month and it became clear that relief was not forthcoming, the attitudes, descriptions, and tone of some other journals also began to change. Rather than describing what is being eaten, diaries shifted focus to the lack of food, especially as the rations dropped. Inglis, for example, stated that “women got three-quarter rations, children half.”^41^ Limited supplies began to exact a toll on the health of the garrison. On the nineteenth of July, Inglis recorded how “the want of change of diet was beginning to be felt, and in addition to other diseases cholera, small-pox, and especially scurvy, began to be fearfully prevalent…Scurvy took the form of loose teeth, swollen heads, and boils, and gained the name of ‘garrison disease’.”^42^

At this point in the siege of Lucknow the relationship between food and medicine became most apparent. Rather than using food to treat or reverse the onset of illness, the diarists recorded the use of food to limit the impact of illness as far as possible. For example, the diarists relied on arrowroot and sago as a means of fortifying themselves, and saved luxury items for the children or the sick, although by this point in the siege the line between sick and healthy was largely arbitrary.^43^ When Inglis went to see Mrs. Radcliffe (a few weeks later):

she was quite lame from what all, more or less, suffered from, boils and eruptions. The slightest scratch inflamed, owing to the bad air and want of vegetable food; and it was on this account that so few who were wounded at all severely re-covered. Amputations were, I believe, with only two exceptions, fatal, and the least wound became serious. The poor sick men, who ought to have had everything that was nourishing and delicate, (have) little else than rations of beef and rum; and…very little of that.^44^

Aside from the relationship between diet and health, Inglis’ account suggests some of the ways in which the garrison actively used what little food they could spare to treat illness. For example, Germon recorded how poultices, typically composed of mustard or attah, were employed for a range of ailments including injuries, diarrhea and boils. They recorded little, however, on whether these cures worked, and Inglis recorded that boils became practically endemic as the extreme lack of food wore on.^45^

Food was also used as a means of treating cases of prolonged illness, such as diarrhea, fever, cholera or in an attempt to reverse the effects of poor diet. Henry Martineau Greenhow, one of the many doctors at Lucknow, reported that “regulation of the diet was, in nearly all instances, a thing difficult to carry out…[but] the indications were to avoid greasy cookery, meat and chuppaties; while rice, soojee [a form of semolina], arrowroot, sago and tea, and subsequently broth, minced-meat and port- wine were allowed. For children, port-wine, soojee or milk and ground rice, with some sugar added, were found to constitute the best diet”.^46^ Greenhow’s ministrations of carbohydrate-rich foodstuffs, while in opposition to earlier medical sources such as Wade, nonetheless appeared to originate from a desire to provide a diet of bland food so as not to upset the stomach. Articles that would take longer to digest might slow regularity of bowel movements. Outside of the garrison, William Russell also wrote of how his servant Simon fed him congee (a form of rice porridge, made from the starchy water in which rice is boiled) when he was ill, though Russell revolted and after taking congee for a day or so decided to switch to “claret and currie”^47^

## Alcohol and Liquid medicines

As Greenhow’s methods, Russell’s efforts at self-medication, and many of the narrative accounts from Lucknow indicate, rarely was food alone used as medicine. Rather, alcoholic and non-alcoholic drinks were consumed throughout the siege as a means of preserving health or remedying illness, and were often highly prized as either medicine or nourishment. Unlike food, which was used to attempt to arrest decline as a result of illness or slow its progress, the consumption of various drinks was often in pursuit of their stimulant properties, despite evidence of medical and social dispute over the benefit of such stimulants to the body. Although the garrison and the medical personnel present would have been aware of this contradiction, it appears they condoned their use in the specific circumstances of a siege of limited duration.

One example is tea.^48^ Tea was available in abundance within the garrison, and, while supplies dwindled in late October towards the very end of the siege, it was one of the few foodstuffs mentioned that was not apparently subject to strict ration.^49^ Aside from the medical use of tea in treating dysentery and diarrhea, it appeared to play a vital role in daily routine at breakfast and aided the psychological, as well as the physiological, health of the diarists. The usefulness of tea in this sense again corresponds to contemporary medical opinion of writers on health and diet in India; Hull noted that tea belongs to a group of:

certain articles of food, which are not particularly nourishing in themselves, but which serve some important purpose in animal economy….they excite the brain, they calm the nervous system generally, and though they produce a state of wakefulness and activity, yet they also induce a species of languor and repose.^50^

Hull’s remarks, however, contrast starkly with the general disposition towards tea in Britain during the same period; Ian Miller notes the disdain for stimulant substances such as tea, coffee and alcohol that “contained no nutritive qualities” and that were considered actively harmful to the British stomach.^51^ In the emergency context of the siege environment though, the need for such stimulants given the drastically reduced intake of other foodstuffs appeared vital, particularly for the men engaged in vigorous physical exercise. The fact that tea was boiled would have also reduced the chances of drinking contaminated water from the garrison’s various wells, and thus offered an indirect benefit to health.^52^ Additionally, that the British at Lucknow anticipated the siege to last only a short time might also have contributed to their disposition towards stimulants like tea and coffee; while excessive or long-term consumption of such drinks was thought to cause nervous excitability and over-stimulation until late into the century, the expectation at Lucknow was for a brief siege followed by relief from troops stationed nearby.^53^ As the siege continued, the consumption of tea contributed to the metanarrative of British endurance that these diaries relate; the drinking of tea often accompanied the reading of the Bible at morning and evening, stimulating the body while religion soothed the mind. Tea was thus linked to connotations of resilience, faith, and fortitude with which the garrison face their situation.^54^

Alcohol, like tea, appeared as part of the daily diet and routine. Alcohol and British India were inherently linked, not only by the EIC trade in various alcoholic beverages such as brandy and arrack, but also through the supply of alcohol given to Company and Crown troops as part of their daily rations. Meanwhile, debate over the efficacy of alcohol in health and medicine for Europeans and Indians occurred throughout the nineteenth century.^55^ The supply of alcohol to troops consisted of a pint of raw spirits per day per man for much of the first half of the century, despite various calls for the ration to be switched to more “wholesome” drinks such as beer.^56^ Although by the 1850s the atmosphere in which later commentators asserted that “heavy drinking, even to the extent of complete drunkenness, was a common thing among all classes” had begun to drop away, widespread alcohol consumption was still very much the norm, and drinking habits in colonial India did not seem divided by gender, with women consuming drinks such as beer, port, and sherry as often as men.^57^

In the diaries from Lucknow, soldiers, male and female civilians and medical men all placed importance on the use of alcohol in treatment, in everyday diet and routine, and as substitute for food during the siege. Recalling the descriptions of dinners by diarists such as Maria Germon, a number of the diarists recorded how their evening meals either consisted of or contained alcoholic drinks.^58^ In addition to the general habit of consuming smaller evening meals, the inclusion of alcohol performed two separate functions. First, it was generally believed that alcohol was an aid to digestion and other complaints of the stomach. The use of alcohol for these purposes was advocated in a number of medical volumes beginning with Wade’s remedies from the 1790s, through those of naval surgeon James Wallace in the early nineteenth century, and later studies by Louis Parkes and Thomas King Chambers in the 1860s and 70s.^59^ There was, of course, opposition to the belief in alcohol’s digestive properties, with a number of tracts and other texts focusing on its deleterious effects published in tandem to those that supported it.^60^ Despite this changeable position, the evidence from medical practice at Lucknow suggests that medical personnel there favored its use and, given the rough and coarse food that was available to the garrison, likely thought alcohol a necessary inclusion in their daily diet. Second, although more rigorous chemical analysis of the component properties of beer and other alcoholic beverages would not take place until much later in the century, there was nonetheless a contemporary recognition of the filling properties of beer and grain used in brewing; in the place of flour and other carbohydrates or starchy foodstuffs, the consumption of alcohol, ale and beer in particular, would have served as a replacement for this lack of nourishment.^61^ Beer had been thought of for centuries as a form of “liquid bread” specifically because it contained the same ingredients: namely yeast, grain, and water.^62^

As with flour, rice and other supplies, many diarists mention that they or their husbands, or others in the garrison had the foresight to gather stores of beer, wine, brandy, sherry, port or champagne before the siege began, and that these were put to various preventative and restorative uses over the course of the ensuing months.^63^ In N.A. Chick’s *Annals of the Indian Rebellion 1857-58* (1858), Brigadier Inglis, recounted how “[w]e possessed some supply of bottled beer. This, which was esteemed the greatest luxury during the siege, had ceased for many days to be served to the gentlemen, and was instead reserved for the nursing ladies…or the sick.”^64^ Inglis’ account of drinking suggests belief in the nutritional or at least nourishing value of beer – that it was reserved for those either already ill or those engaged in vital medical work is indicative of how it was perceived as beneficial to the maintenance of good health, or in bringing an individual back from illness. As the siege progressed, and supplies began to dwindle, alcohol, like food, was subject to a program of rationing, and beer became no longer a dietary supplement but was reserved for those in the most need of it. Germon recorded the ration for twenty people as follows: two bottles of indifferent champagne, one of claret, a pint of sherry and two pints of beer for the sick ladies.^65^ Julia Inglis related how, by the 29^th^ of August: “Spirits, wine, and beer had long run out, except a very little which was kept for the sick.”^66^ If no beer was available, then other fortified drinks were also consumed; Brydon described how William, who was recovering from wounding and illness, received a bottle of brandy which was “so essential to keep up his strength” especially as he was “still so weak, tho’ off the sick list and doing his duty daily”.^67^ The suggestion from these texts is that by the latter stages of the siege, alcohol and beer were no longer reserved for those members of the garrison who would benefit from it in the service of their work, but rather for patients with the greatest need of sustenance.^68^

It was not just lay members of the garrison who condoned the use of alcohol in relation to health and medicine. Greenhow suggested that there was “certainly porter available for those who urgently required it,” and wine and claret made regular appearances in his recommendations for fever and dysentery.^69^ Fayrer related how he too had purchased “all the wine, beer and brandy he could procure” at the outset of the siege, which was then used for its stimulant properties until it began to run low in September.^70^ The belief in porter as a restorative long pre-dated the siege and continued long after, being recommended for convalescents in medical guides, cookery texts, and in lay contexts well into the twentieth century.^71^ However, this belief encapsulates a recurrent contradiction in the medicinal and health-related use of alcohol, namely that it continued to play a central role in recommendations for diet and treatment, despite clear medical knowledge and social evidence of its ill effects. While one might argue that this same charge could be levelled at various foodstuffs at different points during the nineteenth century, none would draw such persistently contentious, or changeable, opinion as alcohol. Hull and Mair, again drawing on the studies of the 1860s and 70s, were predominantly anti-alcohol in their recommendations, yet stated that beer can be “prescribed medically,” and went on to recommend various wines and decoctions for dyspepsia, disease of the liver, and other ailments. Hull also went as far as to provide an overview of the available brands of porter best suited for invalids.^72^ Such a recommendation, and indeed the use of beer throughout the siege, stands in opposition to Reverend Polehampton’s remarks on the 6^th^ of April, 1857 that “unlimited beer and the heat have in consequence increased my fever cases in the hospital.”^73^

Aside from beer, there were more particular uses of alcohol for specific purposes throughout the siege at Lucknow. One infamous example is as a substitute for analgesics. Accounts of the siege written by medical personnel and civilians alike commented often on the lack of supplies in either the European or native hospitals, and Inglis and Case expressed worries over the rapidly decreasing supplies of chloroform for amputations or major surgery. Case described how a Mr. Studdy, too weak for chloroform, was given a bottle of champagne before undergoing amputation of his right arm, and “bore it…without saying a word.”^74^ Likewise, Georgina Harris described the last days of Henry Lawrence, the original garrison commander who lost most of his leg to a round shot early in the siege. Harris had been assigned to nurse him, and described the immense quantity of arrowroot and champagne he had taken, again presumably on medical advice and to dull the pain of his injuries.^75^ The much higher sugar content of nineteenth-century champagne compared to modern varieties might also have contributed to its valued properties as a stimulant.^76^

The other major affliction suffered at Lucknow was the persistent presence of cholera. As a broad range of critical literature indicates, before Louis Pasteur, Robert Koch and the advent of bacteriology in the late 1880s, a range of competing theories and remedies governed Anglo-Indian medical practice around cholera and often combined both food and drink.^77^ For example, an anonymously authored guide entitled *An Anglo-Indian Domestic Sketch* (1849) recommended homemade cholera pills “composed of calomel, opium, asafoetida, camphor and black pepper” as a remedy, though no indication of their reliability was provided.^78^ At Lucknow and elsewhere, cholera was attributed to the foul air caused by rotting animals, bodies, and human waste.^79^ Various approaches to treating cholera were documented in the accounts of the siege, from Germon’s elation at receiving six bottles of mustard (to use in poultices), through to Fayrer’s seeming inability to treat it.^80^ Greenhow provided the most comprehensive overview of treatment, stating that he prescribed a form of cholera pill containing opium and creosote, alongside drinks of congee water and dilute sulphuric acid, while he prohibited the use of stimulants such as alcohol.^81^ Greenhow, incidentally, is one of the few medical accounts to note the beneficial effects of specific foodstuffs on cholera recovery rates, particularly singling out the application of tea, beef-tea, arrowroot, rice, minced meat, and sago.^82^

Such treatments corresponded with contemporary medical thinking on the treatment of cholera.^83^ By the mid-1850s, sources endorsed by the Royal College of Physicians presented four main treatments for cholera infection: the Alterative (comprising mainly of calomel – mercury chloride – doses), the Astringent (including emetics and brandy) the Stimulant (including opium, brandy and mustard coated flannels, or poultices), and the Eliminant or cathartic (which rather vaguely relied on “assisting nature to get rid of the virus”).^84^ The success rates were highly variable, but those involving quantities of brandy, such as the Astringent, were calculated at between 20.3% and 50%, whereas the Eliminant was 71.7%.^85^ However, Greenhow conceded that at Lucknow he could not prevent Europeans taking stimulants to treat cholera. Although he conceded that “Europeans will tolerate it better than natives” due to the fact they were used to it, he was sure that mortality resulted in most instances where alcoholic drinks were administered to cholera patients, leading him to conclude that: “in very few cases is brandy (the best stimulant) advisable even when cholera [or] any other diseases that may prevail.”^86^ In cholera cases at the Native Hospital, Greenhow stated that nearly half recovered.^87^

## Conclusion

In the course of this article I have illustrated the vital role that food and drink played in the diaries and journals produced in the setting of the Indian Rebellion of 1857. The representations of food and drink within these narratives can serve as a lens through which to view the quotidian habits and social composition of British India. The inclusion of food and drink in these texts served a number of purposes. Authors wrote to capture their intimate personal experiences of daily subsistence and their attempts to preserve bodily health or treat illness through regimens of consumption. But they equally constructed narratives to convey a message of individual and national fortitude during British efforts to survive the hardship and deprivations of war. The shortage of food is a tangible source of suffering throughout these accounts, but the efforts to secure particular foodstuffs also demonstrates that these authors (especially those from the upper strata of the colonial society) while they were unaccustomed to the day-to-day realities of cooking and providing for their families, possessed a general elementary knowledge of the importance of nutrition, aided, at least in part, by the concentration of doctors and medical personnel gathered at Lucknow for the duration of the siege. Additionally, the influence of native servants and their shared understanding of what constituted nourishing food cannot be discounted; although some servants abandon the garrison out of an understandable interest in self-preservation, the adaptation of British residents to native foodstuffs throughout the siege suggests trust and reliance on those servants that remained, as well as simple pragmatism given what supplies were available. Men and women unused to cooking their own food (and with little conception of where else to start) attempted to replicate what they would otherwise customarily be served by their household staff.

The uses of alcohol and other stimulants, on the other hand, suggest a very different relationship of consumption to expert and lay knowledge. The prominent role of alcohol in medical practice and in the habits of garrison members reveals an inherent contradiction in the contemporary understanding of drink and its effects. As medical texts and guides from this period note, the deleterious effect of alcohol on the body in tropical climates and in the treatment of diseases such as cholera was widely supported by personal experience; however, in most cases during the siege of Lucknow, this caution was ignored in favor of the strong endorsement of the restorative and stimulant powers of alcohol and of its healthful effects in digestion. Unlike some of the foods described in these accounts, alcohol, and to a similar extent tea, were substances that the British residents were familiar with and, as Greenhow notes, accustomed to. These were used with greater confidence as forms of self-medication. Their particular abundance and long life further contributed to their ready use, especially as supplies of food, fresh or otherwise, began to run out towards the latter months of the siege. Occasionally, however, reading these diaries reveals that, much like the course of the rebellion, the fragile position of the British was exacerbated largely by the actions of the British, with their attitudes to food and drink a similar mixture of unintentional and occasionally willful ignorance, with potentially deadly results.

## Footnotes

1 See Ralph J. Crane, *Inventing India: A History of India in English Language Fiction* (London: Palgrave Macmillan, 1992).

2 Richard Holmes, *Sahib: The British Soldier in India* (London: Harper Perennial, 2006), 252.

3 E. M. Collingham, *Imperial Bodies: The Physical Experience of the Raj, 1800-1947* (London: Polity, 2001), 1. Additionally, Holmes states that for most soldiers in the first half of the nineteenth century a posting to India was a life sentence, as more would die in India than would see their homes again: Holmes, *Sahib*, 233.

4 Mark Harrison, *Climates and Constitutions: Health, Race, Environment and British Imperialism in India 1600-1850* (Oxford: Oxford University Press, 2002), 2, 19-20. See also David Arnold, *Science, Technology & Medicine in Colonial India* (Cambridge: Cambridge University Press, 2000), 76.

5 John Pearman, a sergeant in the King’s Own Light Dragoons, served in India during the 1840s-50s and describes how the men paid natives for various services from laundry to the provision of coffee. Marquess of Anglesey, ed., *Sergeant Pearman's Memoirs* (London: Jonathan Cape, 1968), 27.

6 Arnold, *Science, Technology & Medicine*, 79. See also Mark Jackson, *The Oxford Handbook of the History of Medicine* (Oxford: Oxford University Press, 2011), 294.

7 See Bernard Porter, *The Absent-Minded Imperialists: Empire, Society, and Culture in Britain* (Oxford: University Press, 2004), and Thompson, Andrew, ed., *Britain’s Experience of Empire in the Twentieth Century* (Oxford: Oxford University Press, 2012) for opposing views on the subject.

8 Paul Scott used this phrase, originally attributed to Benjamin Disraeli after Queen Victoria was appointed Empress of India, as the title of the first novel in his *Raj Quartet* series in 1966, later adapted for television in 1984.

9 Holmes, *Sahib*, xxi. Holmes also notes the ratio of European to Indian troops; in 1857, the year of the rebellion, there were 39,800 British troops to 226, 500 Indian (216).

10 There were some precursors such as the Letters of Eliza Fay, published in 1815, which detailed her experiences as a prisoner of Hyder Ali as well as her other travels in India.

11 See Lawrence James, *Raj: The Making and Unmaking of British Indi*a (London: Abacus, 1997), 235.

12 James, *Raj*, 174.

13 Jane Robinson, *Angels of Albion: Women of the Indian Mutiny* (London: Penguin, 1997), 251.

14 Claudia Klaver, “Domesticity under siege: British women and Imperial Crisis at the Siege of Lucknow, 1857,” *Women's Writing* (2001), 28.

15 Klaver, Domesticity under Siege, 27.

16 See Sam Goodman, “Lady Amateurs and Gentleman Professionals; Emergency Nursing in the Indian Mutiny” in Helen Sweet and Sue Hawkins, *Colonial Caring: A History of Colonial and Post-Colonial Nursing* (Manchester: Manchester University Press, 2015).

17 See Leigh Gilmore, *The Limits of Autobiography: Trauma and Testimony* (Ithaca: Cornell University Press, 2001).

18 Graham Huggan calls such literary techniques the “exoticist production of otherness” used to support a solipsistic vision of the “orient” within European literature and culture. See Graham Huggan, *The Post-Colonial Exotic: Marketing the Margins* (London: Routledge, 2001), 13.

19 William Russell, *The Indian Mutiny: A Diary of the Sepoy Rebellion* (London: George Routledge & Sons, 1860), 3.

20 Edmund C. P. Hull, *The European in India: or Anglo-Indian’s Vade-Mecum, with Medical Guide for Anglo-Indians* (London: Henry S. King & Co., 1874), 229-230.

21 John Peter Wade, *Nature and Effects of Emetics, Purgatives, Mercurials and Low Diet in Disorders of Bengal and Similar Latitudes* (London: John Murray, 1793), 27.

22 Hull, *European in India*, 256.

23 See Mark Harrison, *Medicine in an Age of commerce and empire: Britain and its tropical colonies, 1660-1830* (Oxford: Oxford University Press, 2010), 74; Arnold, *Science, Technology & Medicine*, 77.

24 India Office Archives, British Library. Collection 170 Army in India: diet and rations, IOR/L/MIL/7/7666-7672: 1883-1927.

25 Erica Wald, *Vice in the Barracks: Medicine, the Military and the Making of Colonial India, 1780-1868* (London: Palgrave Macmillan, 2014), 127.

26 Brigadier John Inglis’ after-action report lists at least 11 medical personnel in attendance at Lucknow. See N.A. Chick’s *Annals of the Indian Rebellion* (Calcutta: Sanders Cones & Co, 1859), 872.

27 Maria Germon, *Journal of the Siege of Lucknow: An Episode of the Indian Mutiny*, Edited by Michael Edwardes (London: Constable Publishers, 1958), 49.

28 Germon, *Journal*, 58; 65.

29 Julia Inglis. *The Siege of Lucknow* (London: McIlvaine & Co, 1892), 74.

30 Joshua Duke, *Banting in India with Some Remarks on Diet and things in General* (Calcutta/London: Thacker & Spink, 1885), 51. Banting was a form of high-protein, low-carbohydrate diet developed in the 1860s by a British undertaker named William Banting.

31 Inglis, *Siege*, 87. Geoffrey Moore, *The Diary of the Doctor’s Lady* (Bedford: Jaycopy Ltd, 1979), 25. Fayrer also records that a round shot broke the leg of Lieutenant Harmer while a group of officers dined together; Joseph Fayrer, *Recollections of My Life* (London: William Blackwood & Sons, 1900), 176.

32 Collingham, *Imperial Bodies*, 161. Such rigidity would be satirized in twentieth-century productions such as *Carry on Up the Khyber* (1968) in which a group of besieged British residents continue to dine feigning ignorance of the gunfire collapsing the walls and ceilings around them.

33 Hull, *European in India*, 229.

34 Germon, *Journal*, 66.

35 H. S. Polehampton, *A Memoir, Letters and Diary of the Rev. Henry S. Polehampton, M. A*. (London: Richard Bentley, 1858), 345. Not all the women cook for themselves – Maria Germon records how the appropriately named “Mrs Need” the wife and eventual widow of a civilian resident, cooks many of their meals for them; Germon, *Journal*, 83.

36 Moore, *Diary*, 53.

37 Georgina Harris*, A Lady’s Diary of the Siege of Lucknow (written for the perusal of friends at home)* (London: John Murray, 1858), 81. Georgina Harris was the wife of Chaplain James Harris, and equivalent in rank to Mrs. Polehampton.

38 Katherine Bartrum, *A Widow's Reminiscences of the Siege of Lucknow* (London: James Nisbet & Co., 1858), n.p.

39 Bartrum, *Widow’s Reminiscences*, 28.

40 Germon, *Journal*, 108. Germon’s objections seem prompted by personal sensibility rather than general taste, however, and while there is some complaint at its quality (especially the coarse atta flour) there is no mention of British men or women refusing native food elsewhere in these accounts.

41 Inglis, *Siege*, 71. However, Germon states in October that this is further reduced, with women receiving half a man’s allowance, and children as little as 1/6^th^; Germon, *Journal*, 107. As means of comparison, the report written on the diet of the British soldier in 1883-5 by Surgeon General Home states that the daily meat allowance (separate from that of vegetables and other foodstuffs) of would satisfy only a “moderate appetite” providing the whole of it reached the plate, which after cooking it apparently did not. Home also records that this measurement would often include inedible bone, gristle and fat. See India Office Archives, Collection 170 Army in India: Diet and Rations, IOR/L/MIL/7/7666-7672: 1883-1927.

42 Inglis, *Siege*, 80.

43 For example, sugar is carefully preserved, and Case records how a Miss Dickson complains of having to go without in her tea. Adelaide Case, *Day by Day at Lucknow* (London: Richard Bentley, 1858), 61. Georgina Harris meanwhile, writes on the eighteenth of October with more than a little self-satisfaction that “I am quite used now to no sugar, and don't mean ever to take to it again” Harris, *Lady’s Diary*, 141.

44 Inglis, *Siege*, 113. One amputee, Captain Grant, lingered for nine days after losing a hand in a grenade explosion, however, he was an exception and most died within 24 hours.

45 Germon, *Journal*, 81. Inglis, *Siege*, 80.

46 Henry Martineau Greenhow, *Notes, medical and surgical, taken during the late siege of Lucknow* (Alum Bagh: P.M. Craneburgh, Military Orphan Press, 1858), 10.

47 William Russell, *My Diary in India in the Year 1858-59 Vol. I* (London: Routledge, 1860), 381.

48 Tea was of particular importance to the Victorians, and to the imperial economy. Markman Ellis, Richard Coulton and Matthew Mauger describe tea in this period as a “universal social routine” and “ubiquitous marker of national character” with per capita consumption increasing from 1.48 pounds in 1836 to 5.0 pounds by 1885. See Markman Ellis, Richard Coulton and Matthew Mauger, *Empire of Tea: The Asian Leaf that Conquered the World* (London: Reaktion Books, 2015), 221. However, the growing consumption of tea was often seen as addictive, potentially harmful to the nervous system and a possible cause of a hysterical and discontented disposition; see: Ian Miller, *Reforming Food in post-Famine Ireland: Medicine, science and improvement, 1845-1922* (Manchester: University of Manchester Press, 2014).

49 Inglis mentions in passing that tea is rationed (*Siege*, 54), but she is the only diarist who does so; there is a general consensus between the texts though that tea supplies begin to run out after the fifteenth of October, nearly three and a half months into the siege. There are brief mentions of coffee in Case’s diary, mainly to do with how expensive it is when food is auctioned (shillings per pound), but it does not appear that many of the residents had the same supplies of coffee as they did tea.

50 Hull, *European in India*, 234-235. Hull was also the author of *Coffee, Its Physiology, History and Cultivation* (Madras: Adel Press, 1865), and from the introduction to this book, seemingly a planter himself.

51 Ian Miller, *A Modern History of the Stomach* (London: Routledge, 2016), 35.

52 For the importance of boiling water see Alexander Kenny and Major Shelley Leigh Hunt, *Tropical Trials: A Handbook for Women in the Tropics* (London: W.H. Allen, 1883), 129, which speaks to a much longer practice in India of boiling water, or collecting rainwater for drinking; see also, David Burton, *The Raj at Table: A Culinary History of the British in India* (London: Faber & Faber, 1993), 213.

53 Ian Miller, *Reforming Food in Post-Famine Ireland: Medicine, Science and Improvement, 1845-1922* (Manchester: University of Manchester Press, 2014), 96.

54 Tea likewise plays a part in the joy of the relief: Polehampton writes with elation on the nineteenth of November that the soldiers gave her “tea with milk and sugar, and bread and butter, the first we had tasted in months!” Polehampton, *A Memoir*, 351.

55 The EIC ledgers contain a detailed breakdown of quantities of various goods, including brandy and beer, shipped and sold to and from India; See India Office Archives, IOR/L/AG/1/1/9/f.145(1): 1682-1694 as an example. Elsewhere, especially in Britain, the debate over alcohol centered on the actions and reaction to the Temperance Movement and other drives towards public health; for information see James Kneale, “The place of drink: Temperance and the public, 1856–1914,” *Social & Cultural Geography* 2, Issue 1 (2010) 43-59; Harald Fischer-Tiné and Jana Tschurenev, eds., *A History of Alcohol and Drugs in Modern South Asia: Intoxicating Affairs* (London: Routledge, 2014)*;* Harald Fischer-Tiné, “‘The Drinking Habits of Our Countrymen’: European Alcohol Consumption and Colonial Power in British India,” *The Journal of Imperial and Commonwealth History*, 40, no. 3 (2012) 383-408. Indra Munshi Saldanha, “On Drinking and ‘Drunkenness’: History of Liquor in Colonial India,” *Economic and Political Weekly* 30, no. 37 (1995) 2323-2331.

56 India Office Archives: Army, Recruits, Proceeding to India, Beer substituted for spirits and effect reported, IOR/Z/E/4/45/A3018: 1838-1842. The ration was eventually discontinued in the 1880s, however, reinstated in the early decades of the twentieth century for a brief period: Collection 425/1034 Report of court of enquiry into heatstroke cases among troops from Ballarat. IOR/L/MIL/7/18201: 1916-1920.

57 Ethel Landon, *Alcohol: A Menace to India* (Madras: The Christian Literature Society for India, 1918), 7.

58 Colina Brydon writes of how in early October “we had a bottle of beer for dinner” *Diary*, 45.

59 See, James Wallace, *A Voyage to India, containing Reflections on a Voyage to Madras and Bengal in 1821 on the ship Lonach; INSTRUCTIONS FOR THE PRESERVATION OF HEALTH IN INDIAN CLIMATES* [sic] (London: T & G Underwood, 1824). The work of Louis Parkes on alcohol within diet, first published in the 1860s, and that of Thomas Chambers, of the 1870s, condemned excess in alcohol consumption but also actively condoned its use in moderate doses as a digestive aid. See: Louis C. Parkes, *Hygiene and Public Health* (London: H.K. Lewis, 1889). Thomas King Chambers, *A manual of Diet in Health and Disease* (London: Smith, Elder & Co, 1876) 2^nd^ Ed. In spite of the growing medical and scientific opposition to alcohol, in an echo of the debates on cholera (see below) there were still some adherents to older methods and beliefs that lasted into the twentieth century; for example, Patrick Manson states that “doses of alcohol are…decidedly of service”, albeit in strict moderation. Patrick Manson, *Tropical Diseases: A Manual of the Diseases of Warm Climates* (London: Cassell & Company, 1914), 134.

60 See for instance, James Johnson, *Influence of Tropical Climates on European Constitutions* (London: J. J. Stockdale, 1813); Monier Williams,

61 See James Herbert, *The Art of Brewing India Pale Ale & Export Ale, Stock and Mild Ales, Porter and Stout*. (Burton-Upon-Trent, published by the author, 1866), and Charles Simmonds’ *Alcohol: Its Production, Properties, Chemistry, and Industrial Applications* (London: MacMillan & Co, 1919). Similarly, knowledge of beer as a calorific substance had been in circulation since the term’s coinage in the early nineteenth century; see also H.C. Menkel’s *Healthful Diet for India* (Lahore: Civil and Military Gazette Press, 1927).

62 This assessment is still current, and is repeated in Jonathan Hennessy Mike Smith’s *The Comic Book Story of Beer: The World’s Favourite Beverage from 7000 BC to Today’s Craft Brewing Revolution* (Berkeley: Ten Speed Press, 2015), 7.

63 Inglis mentions her own stores, as well as those of Monsieur Deprat, a civilian resident and volunteer. Inglis, *Siege*, 38; 157. Colina Brydon also mentions the Gubbins’ stores and their supplies of alcohol: *Diary*, 49.

64 Chick, *Annals of the Indian Rebellion*, 882.

65 Germon, *Journal*, 94.

66 Inglis, *Siege*, 138.

67 Brydon, *Diary*, 49. Brandy was very strongly associated with India and in use as a general daily beverage mixed with water (“brandy pawnee” or as a “chota peg,” i.e. a small measure) up until the 1870s where it was replaced by gin, after the advent of Schweppes’ anti-malarial tonic. See Richard Barnett, *The Dedalus Book of Gin* (Sawtry: Dedalus, 2011), 135. Case also records how a glass of wine was used to steady the nerves of Mrs Cooper after the explosion of a mine near the ladies’ residence. Case, *Day by Day*, 142.

68 This is corroborated by Greenhow who states that “luxuries” such as “jellies, port-wines, arrowroot, and sago” were sent to the European Hospital; *Notes Medical and Surgical,* 6.

69 Greenhow, *Notes Medical and Surgical*, 4.

70 Fayrer, *Recollections of My Life*, 133; 209

71 John Watney calls attention to a wounded cavalry officer at Waterloo specifically requesting a glass of Guinness porter and ascribing his recovery near entirely to it; see John Watney, *Beer is Best: A History of Beer* (London: Peter Owen, 1974), 116. Similarly, an India Office report from the late 1890s recommends the issue of porter to soldiers’ wives to fortify them on long sea voyages. See Collection 201/26 Proposed daily issue of beer to non-commissioned officers over rank of Sergeant for themselves and their wives. IOR/L/MIL/7/8976: 1897-1898.

72 Hull, *European in India*, 236; 98-99.

73 Polehampton, *Memoir*, 218.

74 Case, *Day by Day*, 138.

75 Harris, *A Lady’s Diary*, 78-79.

76 Champagne was in use medicinally in other contexts; Hull suggests that in the want of a general specific for seasickness, “champagne, Moselle, and sparkling hock” are found very efficacious, and suggests a pint bottle of either would be a valuable acquisition. Hull, *European in India*, 16.

77 Ira Klein, “Cholera: Theory and Treatment in Nineteenth Century India,” *Journal of Indian History* 58 (1980), 40. Klein goes on to argue that the Indian Medical Service remained resistant towards bacteriological pathologies of cholera since so many of its senior officers had openly supported miasma theory. Christopher Hamlin also notes the opposition to Koch’s theories caused by cholera’s persistence in India after it had receded elsewhere. Hamlin notes that cholera was still responsible for 200,000 deaths (per cent of the world total) after the First World War; Christopher Hamlin, *Cholera: The Biography* (Oxford: Oxford University Press, 2008), 207-208.

78 Anonymous, *An Anglo-Indian Domestic Sketch* (Calcutta: W. Thacker & Co, 1849), 140-141. Fairly presciently, the book also recommends collecting rainwater or boiling any drinking water before use.

79 Inglis, *Siege*, 79; Thornhill, *Indian Mutiny*, 265.

80 Germon, *Journal*, 61. Fayrer records numerous cholera cases, but gives little indication of being able to treat them; his later work *Natural History & Epidemiology of Cholera* (London: John Churchill, 1888) suggests he was, like many of the IMS, an advocate of miasma theory, and in a lecture delivered at St. Katherine’s Royal Hospital, London, in 1893 he states he is “unable to accept water theory” as sufficient explanation for cholera outbreaks; “Nursing in Cholera, and how to deal with it in First Aid” (London: Lowe Brothers, 1893), 10.

81 Greenhow, *Notes Medical and Surgical*, 7.

82 Greenhow, *Notes Medical and Surgical*, 10-11.

83 See Mark Harrison’s comprehensive discussion of the debates over the causes, transmission and treatment of cholera in *Climates & Constitutions*, including the generally hostile response to the ideas of John Snow in the 1850s: 179, 191.

84 See: Paris, Alderson, Babington, Tweedie and Bagshaw-Ward, On the Treatment of the Epidemic of Cholera (London: Royal College of Surgeons, 1855); J. S. Pearse and Jeffrey A. Marston, Statistics of the cases of the Cholera Epidemic 1853 *Medical Times and Gazette*, Vols I & II (London: John Churchill, 1854).

85 Report of the Medical Council to the Right Hon. Sir Benjamin Hall in relation to the cholera-epidemic of 1854 (London: George E. Eyre & William Spottiswoode, 1855), 14.

86 Greenhow, *Notes Medical and Surgical*, 7-8.

87 Greenhow, *Notes Medical and Surgical*, 8.

